# Editorial: Integrity of the Autonomic Nervous System in Psychiatric and Neurological Disorders

**DOI:** 10.3389/fneur.2020.00138

**Published:** 2020-02-25

**Authors:** Timo Siepmann, Ben Min-Woo Illigens, Kristian Barlinn

**Affiliations:** ^1^Department of Neurology, University Hospital Carl Gustav Carus, Technische Universität Dresden, Dresden, Germany; ^2^Department of Neurology, Beth Israel Deaconess Medical Center, Harvard Medical School, Boston, MA, United States

**Keywords:** autonomic, psychiatry, neurology, assessment, treatment

The autonomic nervous system is an essential neural control network of the human body that maintains physiologic balance. It regulates function of vital systems such as the cardiovascular system, the gastrointestinal system, and the skin. Autonomic neuronal structures permeate and innervate the entire human organism, managing its autonomic functions largely independent from consciousness and thereby securing its survival. Professor Phillip Low, a pioneer of autonomic neurology, has once referred to the autonomic nervous system as the “Cinderella of Medicine,” a field of science, which attracted distinct interest in the scientific community but was limited by a substantial lack of coherent knowledge (1). When Low and a handful of out-of-the-box-thinking scientists such as Sir Roger Bannister from London, a famous neurologist and record-breaking middle-distance athlete, as well as David Robertson from Nashville, a distinguished neurologist and space physiologist, first embarked on the endeavor to explore the autonomic nervous system in the 1960s and 1970s, they could probably not foresee the future impact of their research. Nowadays, their observations provide a basis for diagnosis and treatment of highly prevalent and debilitating disorders, including metabolic disorders such as diabetes and neurodegenerative disorders such as Parkinson's disease.

Over the past decades, techniques to assess structural and functional integrity of the autonomic nervous system have become paramount in understanding the pathophysiology of these diseases (2–4). Functional integrity of the sudomotor and cardiovascular autonomic nervous system can be tested non-invasively using well-established techniques such as quantitative sudomotor axon-reflex test, heart rate variability assessment and tilt table test (5). Structural integrity of the autonomic nervous system can be assessed using imaging of autonomic cerebral control centers such as the insular cortex as well as by immunohistochemical analysis of small nerve fibers in cutaneous punch biopsies (6, 7). Designing and further advancing these techniques helped improving our pathophysiological understanding of autonomic nervous system disorders and allowed identification of novel diagnostic and therapeutic targets. For example, Parkinson's disease has long been believed to be a primarily central synucleinopathy that affects brain regions of motor control. However, recent research has provided evidence that the peripheral autonomic nervous system is affected by deposition of misfolded alpha-synuclein long before motor control is clinically impaired. In these premotor disease stages the pathological form of alpha-synuclein can be detected in peripheral small autonomic nerve fibers of the skin, introducing a potential target for immunotherapy and other forms of targeted diagnostic and therapeutic approaches (8–10). As part of this article collection, Hong et al. reported an increased risk for atrial fibrillation in Parkinson's disease highlighting the significance of cardiac dysautonomia in these patients. In this population-based study in 15,434 newly diagnosed patients with Parkinson's disease, the authors observed a significant predictive association between atrial fibrillation and Parkinson's disease in premotor and early but not in later disease stages. This observation highlights the potential diagnostic value of atrial fibrillation in prodromal and early Parkinson's disease as well as the potential impact of cardiac dysautonomia on cardiovascular risk in these patients.

While neurodegenerative synucleinopathies have recently been in the spotlight of autonomic neuroscience, autonomic dysfunction can in fact occur in a variety of neurological disorders such as diabetic or amyloidosis-related neuropathies, acute ischemic stroke, multiple sclerosis, neuroinflammatory diseases as well as psychiatric disorders such as anxiety and depression. This is of high clinical relevance as autonomic dysfunction can reduce quality of life, increase mortality, and increase cardiovascular risk. For example, autonomic impairment has been shown to independently increase mortality in patients with diabetic autonomic neuropathy and increase risk for cardiovascular disease in patients with depression (11, 12). Moreover, autonomic dysfunction seems to be associated with cognitive impairment as reported by Forte et al. in their systematic review, which is part of this article collection. In 20 studies comprising data from 19,431 study participants, they found that both increased sympathetic activity and decreased parasympathetic activity are associated with cognitive impairment. Notably, in the majority of included studies these associations were independent from demographic and clinical characteristics supporting a direct link between impairment of autonomic and cognitive functional integrity. Viewed in conjunction with the complex etiopathogenesis of autonomic dysfunction these observations highlight the need for personalized diagnostic and therapeutic strategies for disorders of the autonomic nervous system. Possible keys to improve early detection and personalized treatment of autonomic dysfunction comprise interdisciplinary symptom-driven clinical management strategies, advancement of assessment and further elucidation of the pathophysiological pathways leading to dysautonomia. Thus, interdisciplinary research on the autonomic nervous system has the potential to help improve quality of life, reduce mortality, and improve cardiovascular health. This would have implications for diseases that extend far beyond classic autonomic disorders such as diabetic neuropathy. Our article collection aims to provide a platform to foster autonomic neuroscience.

## Author Contributions

TS drafted the first version of the manuscript. BM-W and KB revised the manuscript for intellectual content.

### Conflict of Interest

The authors declare that the research was conducted in the absence of any commercial or financial relationships that could be construed as a potential conflict of interest.
